# Comparing differences in onset and clinical manifestations between children and adults with *Mycoplasma pneumoniae* pneumonia and analyzing risk factors for severe cases, during the post-COVID-19 pandemic era

**DOI:** 10.3389/fpubh.2026.1771434

**Published:** 2026-06-12

**Authors:** Yuchen Peng, Liping Liu, Xiaopeng Li, Xiaoping Wu

**Affiliations:** 1 Department of Infectious Diseases, the First Affiliated Hospital of Nanchang University, Nanchang, China; 2 Department of Oncology, the First Affiliated Hospital of Nanchang University, Nanchang, China

**Keywords:** adult, children, clinical classifications, *Mycoplasma pneumoniae* pneumonia, propensity score matching

## Abstract

**Introduction:**

In the post-pandemic era, *Mycoplasma pneumoniae* pneumonia (MPP) has become increasingly prevalent among adults, a population previously considered less affected and poorly studied. This study aimed to compare the clinical features of MPP in children and adults and identify predictors of severe disease.

**Methodology:**

288 MPP patients were retrospectively analyzed. Differences in clinical classification, symptoms, imaging, laboratory markers, and treatment response were assessed between children and adults. Propensity score matching (PSM) was applied to minimize bias. Predictors of severe MPP were further analyzed.

**Results:**

Most cases were mild with predominant respiratory symptoms. After PSM, “Child” had higher thermal spike (39.01 °C vs. 38.66 °C, *p* = 0.017), lymphocyte percentage, and lactate dehydrogenase. “Adult” had higher C-reactive protein (CRP), erythrocyte sedimentation rate. Adults recovered significantly faster, with a higher cumulative improvement rate from day 2 (HR: 1.916, 95% CI: 1.267–2.899). Key predictors for severe MPP diverged between groups. The combination of elevated CRP, reduced lymphocyte percentage, and pre-existing diseases showed good predictive performance (AUC: 0.744) in “Child,” whereas in “Adult,” elevated CRP alone was the sole independent predictor (AUC: 0.749).

**Conclusion:**

Cough is the dominant symptom in both groups. CRP, lymphocyte percentage, and comorbidities help assess severity in “Child”. Elevated CRP alone predicts severity in “Adult”. Quinolones were associated with favorable recovery in adults.

## Introduction

*Mycoplasma pneumoniae* (MP) is a Gram-negative bacterium characterized by the absence of a cell wall (1). It is one of the most common pathogens of community-acquired pneumonia (CAP), accounting for 11.1–38.9% of adult CAP cases (2–4) and is also a significant pathogen in pediatric CAP (5). MP causes disease through mechanisms such as direct damage and immune mediation (6). Although the pathogenesis is generally consistent, the clinical features of mycoplasma pneumoniae pneumonia (MPP) differ between children and adults, with some patients more prone to developing severe cases. Enhanced public health measures (PHM) during the COVID-19 pandemic, such as non-pharmaceutical interventions, timely disinfection, travel restrictions, regional lockdown (7). However, reduced population exposure led to diminished immune priming, with these measures were relaxed or canceled, resulting in a significant increase in respiratory infections compared to pre-pandemic levels (8). Notably, MPP, which were previously uncommon in adults, have now emerged with an upward epidemiological trend.

Our aim was to investigate the clinical characteristics, auxiliary examination features, and clinical outcomes of MPP children compared to adult patients, and to identify factors influencing the progression to severe cases, during the post-COVID-19 pandemic era.

## Methodology

### Study population

The study consisted of patients who were admitted to the First Affiliated Hospital of Nanchang University, with MPP, between September 1, 2023-February 29, 2024. These patients were divided into 2 groups, based on their ages: “Child,” defined as individuals 0–17 years old, and “Adult,” defined as individuals ≥18 years. Patient inclusion criteria were as follows: Patients met the diagnostic criteria for MPP as per the “Guidelines for Diagnosis and Treatment of *Mycoplasma pneumoniae* Pneumonia in Children” (9).

### Clinical classification of MPP

According to the above mentioned guideline, pediatric MPP patients were classified into mild and severe types. Adult clinical classification is generally consistent with pediatric criteria as per the “Guidelines for Diagnosis and Treatment of Community-Acquired Pneumonia in Adults” (10). The detailed severity classification criteria, as defined by the relevant guidelines, are provided in the Supplementary material.

### Data collection and classification of sub-groups

Clinical data, laboratory indicators, chest CT for both Child and Adult groups were collected. Both “Child” and “Adult” groups were divided into 3 age-based sub-groups each: 0–6 years, 7–12 years, 13–17 years for the former; and 18–40 years, 41–60 years, ≥61 years. By MPP severity, patients were divided into Mild and Severe. Antibiotic selection followed relevant national guidelines. Treatment allocation was not randomized and was influenced by age, disease severity, and clinician judgment.

### Statistical analysis

The χ2 test or the t-test was used to assess significant as sociations between exposure and outcome variable. In the case–control analysis, univariable and multivariable logistic regression models were constructed (backward LR). Factors in univariable logistic regression models with *p* < 0.10 were included in the multivariate statistical analysis. Propensity score matching (PSM) was used to adjust for demographic differences, with a caliper value of 0.05 and a 3:2 nearest-neighbor matching method. Kaplan–Meier analysis was used to assess cumulative improvement rates between groups, and comparisons were made using the Log-Rank test. *p* < 0.05 was considered statistically significant. Predictive performance was assessed using ROC curves.

## Results

### Patient characteristics and clinical classifications among different sub-categories within “Child” and “Adult” groups

In the “Child” group, the age of onset ranged from 18 days to 17 years. In the “Adult” group, the age of onset ranged from 18 to 76 years. Clinical classification: In the “Child” group, 170 cases (85.00%) were mild, and 30 cases (15.00%) were severe; in the “Adult” group, 83 cases (92.05%) were mild, and 5 cases (7.95%) were severe. A history of pre-existing disease was present for 10 cases (5%) in “Child,” and 24 (27.3%) in “Adult.” The proportion of different age groups and the IgM positivity rate in adults across clinical classifications (*p* < 0.05) (Table 1).

**Table 1:** Comparing clinical classifications between different “Child” and “Adult” sub-groups.

“Child” sub-group	Mild (*n* = 170)	Severe (*n* = 30)	χ^2^	*p* value
Age, *N* (%)			14.466	0.001
0–6	59 (34.71)	10 (33.33)		
7–12	89 (52.35)	8 (26.67)		
13–17	22 (12.94)	12 (40.00)		
Gender, *N* (%)			0.990	0.320
Male	96 (56.47)	14 (46.67)		
Female	74 (43.53)	16 (53.33)		
Pre-existing diseases, *N* (%)			2.514	0.113
Yes	6 (3.53)	4 (13.33)		
No	164 (96.47)	26 (86.67)		
Positive rate, *N* (%)				
IgM	84 (49.41)	15 (50.00)	0.004	0.953
DNA	146 (85.88)	22 (73.33)	2.988	0.084

“Adult” sub-group	Mild (*n* = 69)	Severe (*n* = 19)	χ^2^	*p* value
Age, *N* (%)			2.474	0.290
18–40	42 (60.87)	12 (63.16)		
41–60	20 (28.99)	4 (21.05)		
≥61	7 (10.14)	3 (15.79)		
Gender, *N* (%)			0.046	0.830
Male	26 (37.68)	8 (42.11)		
Female	43 (62.32)	11 (57.89)		
Pre-existing diseases, *N* (%)			0.018	0.894
Yes	19 (27.54)	5 (26.32)		
No	50 (72.46)	14 (73.68)		
Positive rate, *N* (%)				
IgM	32 (46.38)	8 (42.11)	4.840	0.028
DNA	36 (52.17)	33 (173.68)	2.069	0.150

### Comparing MPP symptoms, laboratory indices and imaging changes between “Child” and “Adult” groups before and after PSM

“Adult” had a higher proportion of females and a higher proportion of patients with underlying diseases compared to “Child” (*p* < 0.05). The main symptoms in “Child” were cough and fever, with the average peak temperature higher than that in “Adult” (*p* < 0.05). The incidences of fever, nasal congestion and runny nose, and nausea and vomiting were higher in “Child” than in “Adult” (*p* < 0.05). On the other hand, among “Adult,” the main symptoms were cough and sputum production, with higher incidences of sputum production, chest tightness and chest pain, throat discomfort, and muscle soreness compared to “Child” (*p* < 0.05). With a higher proportion of severe cases and DNA positivity rates among “Child” (*p* < 0.05). The incidence of ground-glass opacity and small nodules was higher in “Adult” compared to “Child” (*p* < 0.05). After PSM, there were no statistically significant differences between the two groups except for peak temperature (*p* < 0.05). For the matched data, the median C-reactive protein (CRP), erythrocyte sedimentation rate (ESR), and lactate dehydrogenase (LDH) levels were above normal ranges in both groups. The lymphocyte percentage was below normal among “Adult,” while the neutrophil percentage and D-dimer levels were above normal. “Child” with higher lymphocyte percentage, LDH, and PCT levels; CRP and ESR levels were lower among “Child” (*p* < 0.05) (Table 2).

**Table 2:** Comparing MPP symptoms, laboratory indices and imaging changes between “Child” and “Adult” groups before and after PSM.

Variable	Before PSM				After PSM			
	Child (*n* = 200)	Adult (*n* = 88)	χ^2^/Z	*p value*	Child (*n* = 69)	Adult (*n* = 46)	χ^2^/Z	*p value*
Gender, *N* (%)			6.545	0.011			0.983	0.321
Male	110 (55.00%)	34 (38.64%)			35 (50.72%)	19 (41.30%)		
Female	90 (45.00%)	54 (61.36%)			34 (49.28%)	27 (58.70%)		
Pre-existing diseases, N (%)			29.117	<0.001			0.014	0.904
Yes	10 (5.00%)	24 (27.27%)			8 (11.59%)	5 (10.87%)		
No	190 (95.00%)	64 (72.73%)			61 (88.41%)	41 (89.13%)		
Symptoms, *N* (%)								
Fever	183 (91.50%)	70 (79.55%)	8.181	0.004	61 (88.41%)	40 (86.96%)	0.054	0.816
Chills	21 (10.50%)	6 (6.82%)	0.975	0.323	3 (4.35%)	4 (8.70%)	0.913	0.339
Fatigue	5 (2.50%)	11 (12.50%)	11.647	<0.001	3 (4.35%)	6 (13.04%)	2.893	0.089
Cough	199 (99.50%)	85 (96.59%)	3.776	0.052	68 (98.55%)	45 (97.83%)	0.085	0.771
Expectoration	117 (58.50%)	71 (80.68%)	13.266	<0.001	56 (81.16%)	35 (76.09%)	0.430	0.512
Chest tightness/shortness of breath	15 (7.50%)	30 (34.09%)	32.776	<0.001	10 (14.49%)	12 (26.09%)	2.398	0.121
Sore throat (hoarseness)	8 (4.00%)	11 (12.50%)	7.165	0.007	5 (7.25%)	2 (4.35%)	0.406	0.524
Nasal congestion/runny nose	28 (14.00%)	2 (2.27%)	9.007	0.003	2 (2.90%)	2 (4.35%)	0.173	0.678
Nausea/vomiting	32 (16.00%)	3 (3.41%)	9.075	0.003	4 (5.80%)	2 (4.35%)	0.117	0.732
Headache	27 (13.50%)	20 (22.73%)	3.810	0.051	14 (20.29%)	9 (19.57%)	0.009	0.924
Abdominal pain/diarrhea	19 (9.50%)	3 (3.41%)	3.213	0.073	2 (2.90%)	3 (6.52%)	0.871	0.351
Myalgia/arthralgia	3 (1.50%)	6 (6.82%)	5.709	0.017	2 (2.90%)	2 (4.35%)	0.173	0.678
Thermal spike (°C)	39.14 ± 0.75	38.68 ± 0.78	4.344	0.000	39.01 ± 0.69	38.66 ± 0.76	2.421	0.017
Clinical classification, *N* (%)			4.970	0.026			0.151	0.698
Mild	170 (85.00%)	83 (92.05%)			63 (91.30%)	41 (89.13%)		
Severe	30 (15.00%)	5 (7.95%)			6 (8.70%)	5 (10.87%)		
Laboratory indices								
WBC, x10^9^/L	7.14 (6.05, 9.05)	7.28 (4.78, 10.60)	2.488	0.111	7.20 (6.07, 9.01)	9.63 (4.83, 12.93)	1.026	0.307
LYM, %	23.70 (18.40, 30.90)	19.00 (14.85, 25.50)	39.942	<0.001	22.85 (18.25, 30.40)	12.95 (8.55, 17.88)	3.580	0.001
N, %	66.70 (58.20, 73.30)	72.40 (65.95, 77.25)	38.317	<0.001	69.20 (58.13, 74.80)	80.85 (74.40, 85.70)	0.462	0.645
NLR	3.46 ± 2.75	4.70 ± 3.57	4.657	<0.001	3.53 ± 3.26	7.83 ± 4.70	2.027	0.045
CRP, mg/L	14.80 (5.61, 33.80)	28.74 (6.85, 56.47)	40.165	<0.001	17.62 (4.59, 49.16)	50.10 (12.40, 151.04)	5.528	<0.001
PCT, ng/ml	0.16 (0.10, 0.23)	0.22 (0.10, 0.23)	1.316	0.251	0.16 (0.10, 0.23)	0.13 (0.10, 0.22)	0.795	0.428
ESR, mm/h	30.81 (30.80, 46.35)	30.80 (30.79, 46.18)	4.892	0.027	30.81 (30.80, 46.35)	40.68 (30.81, 51.34)	5.223	<0.001
LDH, IU/L	281.70 (249.41, 335.00)	308.00 (249.90, 363.30)	44.601	<0.001	272.00 (249.41, 326.30)	262.90 (229.43, 323.43)	4.466	<0.001
D-dimer, mg/L	0.52 (0.24, 0.78)	0.54 (0.26, 0.78)	12.284	<0.001	0.38 (0.17, 0.78)	0.65 (0.19, 0.98)	1.699	0.092
CT imaging features, *N* (%)								
Tree-in-bud pattern	1 (0.50%)	3 (3.41%)	3.776	0.052	1 (1.45%)	2 (4.26%)	0.874	0.350
Cumulative double lungs	104 (52.00%)	41 (46.59%)	0.715	0.398	33 (47.83%)	18 (38.30%)	1.030	0.310
Pulmonary consolidation	29 (14.50%)	10 (11.36%)	0.513	0.474	9 (13.04%)	5 (10.64%)	0.152	0.696
Ground-glass opacity	11 (5.50%)	17 (19.32%)	13.295	<0.001	6 (8.70%)	9 (19.15%)	2.713	0.100
Spots/patches	141 (70.50%)	63 (71.59%)	0.035	0.851	49 (71.01%)	37 (78.72%)	0.867	0.352
Strip-like patterns	6 (3.00%)	6 (6.82%)	2.231	0.135	0 (0.00%)	4 (8.51%)	6.082	0.431
Hydrothorax	9 (4.50%)	7 (7.95%)	1.390	0.238	1 (1.45%)	4 (8.51%)	3.380	0.066
Lung mini-nodules	20 (10.00%)	24 (27.27%)	14.086	<0.001	8 (11.59%)	11 (23.40%)	2.847	0.092
Positive rate, *N* (%)								
IgM	99 (49.50%)	40 (45.45%)	0.401	0.527	26 (37.68%)	19 (41.30%)	0.152	0.697
DNA	168 (84.00%)	48 (54.55%)	28.276	<0.001	53 (76.81%)	30 (65.22%)	1.847	0.174

WBC, white blood cell; LYM, lymphocyte percentage; N, neutrophilic granulocyte percentage; NLR, neutrophil To lymphocyte ratio; CRP, C-reactive protein; PCT, procalcitonin; ESR, erythrocyte sedimentation rate; LDH, lactate dehydrogenase; PT, prothrombin time.

### Comparing cumulative improvement rates between “Child” and “Adult” groups after PSM

**Figure 1:**
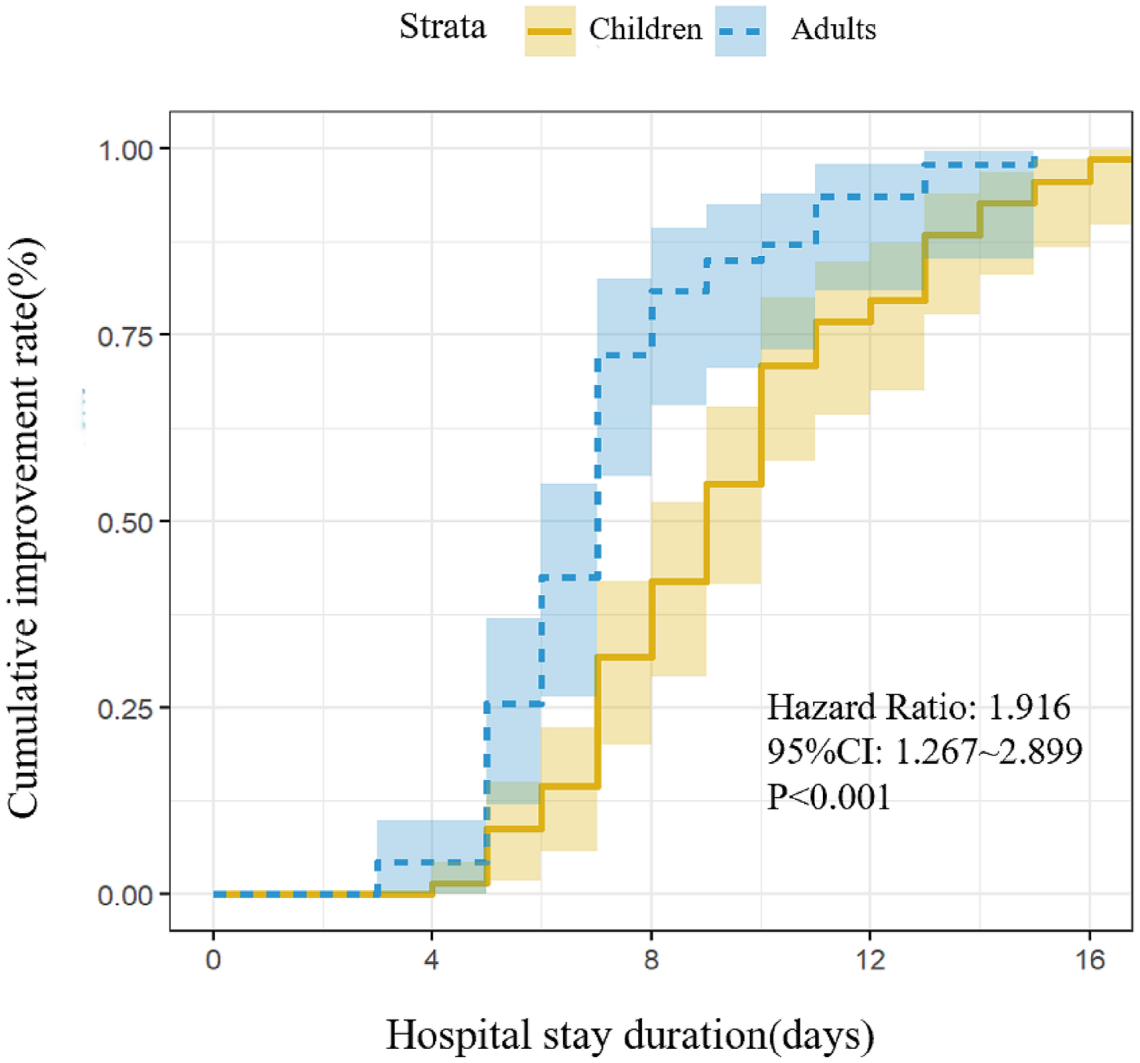
Cumulative improvement rate among “Child” and “Adult” groups after propensity score matching.

The hospitalization duration was longer in “Child” than in “Adult” (*p* < 0.05). The improvement rate was significantly higher in “Adult” compared to “Child” (HR: 1.916, 95% CI: 1.267–2.899, *p* < 0.001) (Figure 1). After PSM, the cumulative improvement rate in “Adult” was higher than that in “Child” from day 2 to day 13 of hospitalization (*p* < 0.05) (Supplementary Table 1).

### Comparing treatments in both “Child” and “Adult” groups

In “Child,” azithromycin was the most commonly used antibiotic. In “Adult,” quinolones were the most frequently used antibiotics and were also linked to the shortest hospitalization duration, although the difference was not statistically significant (*p* > 0.05) (Table 3).

**Table 3:** Comparing treatments in both “Child” and “Adult” groups.

“Child” sub-group	Proportion of drug use *N* (%)	length of stay (Days)	F	*p* value
Mild, *N* (%)			0.326	0.722
Azithromycin	136 (80.00)	9.53 ± 2.92		
Doxycycline	20 (11.76)	9.85 ± 2.54		
Omadacycline	14 (8.24)	9.00 ± 2.77		
Severe, *N* (%)			0.019	0981
Azithromycin	26 (86.67)	11.15 ± 4.21		
Doxycycline	2 (6.67)	11.00 ± 2.83		
Omadacycline	2 (6.67)	9.50 ± 3.54		

“Adult” sub-group	Proportion of drug use *N* (%)	Length of stay (Days)	F	*p* value
Mild, *N* (%)			1.412	0.251
Azithromycin	3 (4.35)	7.33 ± 1.53		
Quinolones	38 (55.07)	7.79 ± 2.41		
Omadacycline	28 (40.58)	6.86 ± 2.00		
Severe, *N* (%)			0.304	0.589
Azithromycin	0 (0.00)	—		
Quinolones	13 (68.42)	7.64 ± 3.65		
Omadacycline	6 (31.58)	7.00 ± 1.58		

### Analyzing factors influencing severe cases

Based on clinical classification, patients in each group were divided into mild and severe groups. Variables with a *p*-value < 0.1 in univariate analysis were included in the multivariate logistic regression. In “Child,” multivariate analysis identified pre-existing diseases (*p* = 0.027), LYM percentage (*p* = 0.017), CRP (*p* = 0.044) as independent risk factors for progression to severe MPP. In “Adult,” CRP (*p* = 0.014) was the only independent predictor of severity (Table 4; Supplementary Table 2).

**Table 4:** Analyzing factors influencing severe cases in “Child” and “Adult” groups.

Variable	Child				Adult			
	Mild (*n* = 170)	Severe (*n* = 30)	Univariate analysis *p* value	Multivariate analysis *p* value	Mild (*n* = 69)	Severe (*n* = 19)	Univariate analysis *p* value	Multivariate analysis *p* value
Age, years	7.02 (4.55, 9.05)	7.07 (5.02, 15.25)	0.009	0.890	35.00 (27.00, 50.00)	31.00 (27.00, 50.00)	0.880	
Male, *N* (%)	96 (50.53)	14 (46.67)	0.320		26 (37.68)	8 (42.11)	0.726	
BMI	15.61 (14.19, 17.65)	16.84 (14.76, 19.14)	0.370		22.05 ± 3.29	21.61 ± 2.48	0.319	
Pre-existing diseases, *N* (%)	6 (3.53)	4 (13.33)	0.023	0.027	19 (27.54)	5 (26.32)	0.916	
Thermal spike, °C	39.00 (38.60, 39.40)	40.00 (39.58, 40.50)	<0.001	0.508	38.90 (38.00, 39.10)	38.50 (38.00, 39.55)	0.865	
WBC, x10^9^/L	7.04 (6.00, 8.89)	7.14 (4.71, 8.72)	0.964		8.26 (6.05, 9.57)	7.73 (5.40, 11.69)	0.611	
LYM, %	26.20 (21.25, 33.05)	20.75 (15.93, 25.93)	0.033	0.017	19.00 (13.40, 22.10)	14.60 (10.10, 26.40)	0.561	
*N*,%	64.00 (56.30, 70.45)	70.15 (63.68, 75.33)	0.434		72.34 (68.60, 78.10)	75.90 (63.40, 81.80)	0.721	
NLR	2.73 (1.79, 3.19)	3.27 (2.19, 4.67)	0.088	0.123	4.08 (3.04, 5.55)	4.15 (2.47, 8.06)	0.121	
CRP, mg/L	9.16 (4.40, 18.65)	23.02 (6.37, 46.57)	0.002	0.044	40.36 (13.58, 58.05)	68.00 (47.55, 100.19)	<0.001	0.014
ALT, IU/L	13.50 (11.00, 18.00)	14.20 (12.00, 22.45)	0.354		26.20 (13.80, 44.90)	24.00 (18.25, 38.60)	0.519	
AST, IU/L	28.00 (23.00, 32.65)	28.40 (22.00, 31.35)	0.647		23.00 (18.50, 35.20)	27.00 (20.10, 33.85)	0.362	
Albumin, g/L	41.00 (38.75, 42.80)	41.35 (39.18, 42.80)	0.021	0.319	40.12 ± 3.65	40.36 ± 6.14	0.904	
Cr, μmol/L	40.20 (34.35, 46.25)	44.26 (37.65, 54.23)	0.371		60.50 (53.10, 74.40)	57.70 (52.50, 68.60)	0.237	
PCT, ng/ml	0.20 (0.10, 0.23)	0.23 (0.10, 0.23)	0.829		0.16 (0.10, 0.16)	0.10 (0.10, 0.16)	0.240	
ESR, mm/h	30.81 (30.80, 30.81)	30.81 (24.25, 31.10)	0.351		46.35 (46.35, 46.36)	46.35 (37.18, 47.18)	0.362	
LDH, IU/L	306.30 (270.10, 351.90)	317.50 (266.08, 377.78)	0.147		249.41 (226.90, 281.60)	249.41 (195.95, 268.70)	0.315	
D-dimer, mg/L	0.78 (0.28, 0.78)	0.45 (0.26, 0.78)	0.899		0.34 (0.17, 0.61)	0.36 (0.18, 0.70)	0.056	0.416
PT, s	12.60 (12.20, 12.70)	12.60 (12.20, 13.65)	0.162		12.20 (11.50, 12.80)	12.30 (11.65, 12.60)	0.437	
Tree-in-bud pattern, *N* (%)	1 (0.59)	0 (0.00)	0.674		2 (10.53)	1 (1.45)	0.615	
Cumulative double lungs, *N* (%)	85 (50.00)	19 (63.33)	0.178		30 (43.48)	11 (15.94)	0.265	
Pulmonary consolidation, *N* (%)	24 (14.12)	5 (16.67)	0.715		8 (11.59)	2 (2.90)	0.897	
Ground-glass opacity, *N* (%)	11 (6.47)	0 (0.00)	0.152		14 (20.29)	3 (4.35)	0.660	
Spots/patches, *N* (%)	119 (70.00)	22 (73.33)	0.712		49 (71.01)	14 (20.29)	0.819	
Strip-like patterns, *N* (%)	4 (2.35)	2 (6.67)	0.202		5 (7.25)	1 (1.45)	0.761	
Hydrothorax, *N* (%)	7 (4.12)	2 (6.67)	0.535		4 (5.80)	3 (4.35)	0.154	
Lung mini-nodules, *N* (%)	19 (11.18)	1 (3.33)	0.187		19 (27.54)	5 (7.25)	0.916	

**Figure 2:**
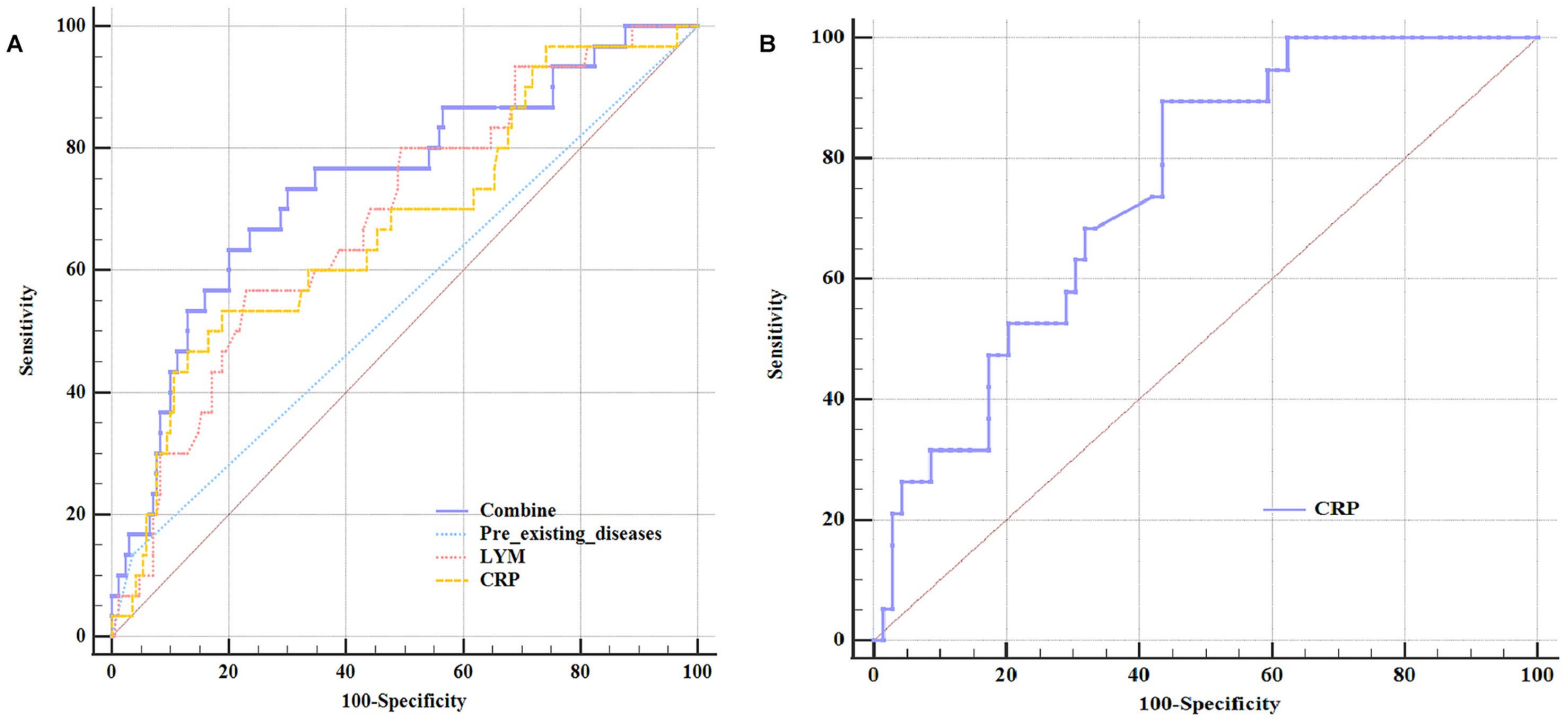
ROC analysis of predictors for developing severe cases. **(A)** ROC analysis of predictors for developing severe cases in “Child.” **(B)** ROC analysis of predictors for developing severe cases in “Adult”.

**Figure 3:**
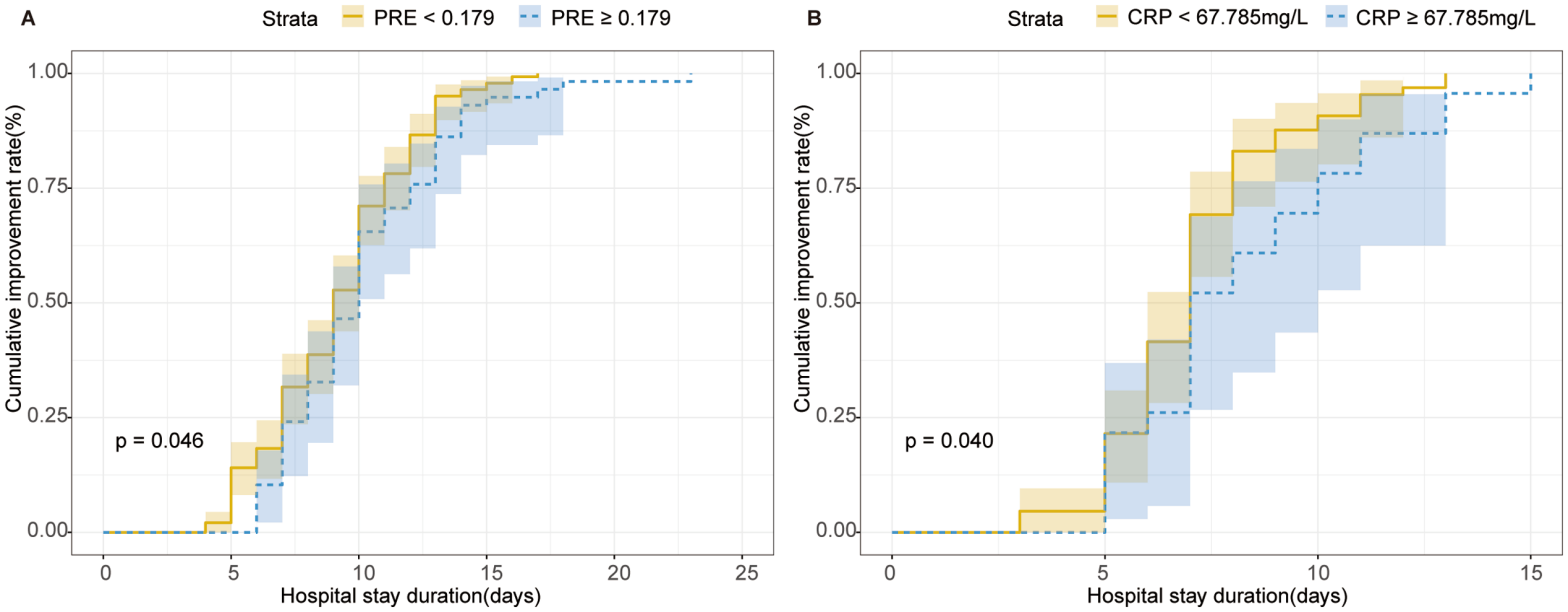
Stratified Kaplan–Meier analysis of time to discharge based on predicted risk. **(A)** In “Child,” patients were stratified into high-risk and low-risk groups using the optimal cutoff value of the combined predicted probability derived from the multivariate model (incorporating CRP, lymphocyte percentage, and pre-existing diseases). **(B)** In “Adult,” patients were stratified by the optimal CRP cutoff value.

To evaluate the predictive performance of the independent factors for severe MPP, receiver operating characteristic (ROC) curve analysis was performed. In “Child” group, the combination of reduced lymphocyte percentage, elevated CRP, and pre-existing diseases yielded an area under the curve (AUC) of 0.744 (95% CI: 0.677–0.803, *p* < 0.001), with an optimal cutoff value of 0.179, a sensitivity of 63.30%, and a specificity of 80.00% (Figure 2A). In “Adult” group, elevated CRP alone was predictive of severe disease, with an AUC of 0.749 (95% CI: 0.645–0.835, p < 0.001), an optimal cutoff of 67.785 mg/L, a sensitivity of 89.50%, and a specificity of 56.52% (Figure 2B). We performed a stratified Kaplan–Meier analysis of time to discharge, with stratification based on cutoff value (Figure 3).

## Discussion

MP characterized by the absence of a cell wall, making it inherently resistant to antibiotics that target the cell wall (1). Its asymmetric morphology features one end of the cell membrane extending outward to form adhesion organelles, which attach to the respiratory epithelium. This attachment is the primary pathogenic process of MPP (11). Despite the structural and pathogenic mechanism consistency of MP, the clinical features of infection differ between children and adults. In the post-COVID-19 era, widespread lifting of public health restrictions has led to increased susceptibility, likely due to attenuated population immunity from reduced environmental exposure during containment (7, 8). As a result, MPP, once uncommon in adults, has shown a marked rise in incidence in this population. Furthermore, some patients are more prone to severe cases. In this study, the severity rates observed was 15% in children and 7.95% in adults. Same as in Europe, data indicated increased severity of MP infections in the latest outbreak, especially in adults, compared to pre-COVID-19 levels (12). A multicenter analysis in China documented MP infection rates rebounding from 1.69% during the pandemic to 19.4–35.9% in 2023–2024 (13). Predictive factors or biomarkers are still being sought.

In this study, indicate that school-age children have the highest proportion of MPP. A study in the United States shows that MP is easily transmitted through droplets and direct contact. During school periods, dense populations lead to an increased proportion of infections among school-age children. Previous studies have shown that pneumonia caused by MP infection is uncommon in children under 5 years of age, and the incidence is highest in school-age children between 5 and 15 years of age (14). However, in this study, preschool children also constituted a significant proportion of MPP, with many developing severe cases. Studies have shown that MP possesses unique terminal organelles that mediate cell adhesion. These organelles tightly bind to the sialylated glycoprotein receptors on the host’s mucosal epithelial cells through membrane adhesins (including P1, P30, P116, and HMW1-3), resisting mucociliary clearance and phagocytosis. This enables MP to inject various hydrolases into host cells, causing localized respiratory tract damage (15). Preschool children have underdeveloped organs, poor ciliary movement, smaller lung structures, and relatively narrow airways, making them more susceptible to MP infections and more likely to develop severe cases post-infection (6). The finding that young and middle-aged adults have a higher proportion of MPP was in line with observations from Ren et al. (16), likely due to their presence in educational or workplace environments, which are more densely populated and involve more frequent social interactions.

Patients in this study primarily presented with symptoms of upper respiratory tract infection such as fever, cough, and sputum production. The proportion of children with fever was higher than that of adults, and their fever peaks were higher. A study by Appalachian State University indicated that children have lower cardiac output and sweating rates compared to adults, resulting in poorer thermoregulatory responses (17). The proportion of adults with sputum production was higher than that of children. Children’s narrower respiratory tracts, delicate mucous membranes, and poorer ciliary movement make it difficult for them to expel sputum effectively and clear invading bacteria. The proliferation of bacteria leads to mucosal edema and local inflammatory responses, which, if not treated promptly, can cause respiratory distress and accelerate disease progression (18). This contributes to the higher incidence of severe cases in children compared to adults. The pronounced tendency of children to develop severe MPP compared to adults can be partly explained by age-dependent divergence in both innate and adaptive immune responses. Toll-like receptors (TLRs) are a crucial component of the innate immune system, playing a key role in pathogen recognition and immune response initiation (19). TLRs levels are lower in children, whereas adults have higher levels, enabling early recognition of MP. This early activation of the immune system helps delay disease progression and reduces the incidence of severe MPP (20). Moreover, the adaptive immune system in children is still maturing, with a bias toward a Th2-dominant or less focused Th1 response, which may impair effective clearance of intracellular or cell-wall-deficient pathogens like MP and simultaneously promote immune-mediated tissue injury (21, 22).

When MPP involves the alveoli, small amounts of serous and inflammatory exudates accumulate in the alveolar cavities, leading to thickening of the septa and partial alveolar atrophy. In more severe cases, fibrous exudation and the formation of hyaline membranes can occur. CT imaging shows ground-glass opacities, appearing as indistinct, cloud-like shadows that do not completely obscure the underlying lung markings. These findings are more common in adult MPP (23). In this study, ground-glass opacities were more common in adult patients than in children. The relatively lower number of alveoli and the incomplete development of lung physiology and function in children may explain the higher proportion of severe cases but fewer changes in ground-glass opacities compared to adults.

After PSM adjustment, the differences in general clinical data between the two groups of patients were minimized. Comparing the laboratory indicators between the two groups after PSM, lymphocytopenia was more common in adults than in children. MP infection triggers a complex lymphocytic response: on one hand, previous research has shown that MP can not only adhere to the host cell surface but also exhibit gliding motility, which is why it is referred to as “walking pneumonia (11, 15)”. During gliding, it repeatedly captures and releases surface structures, consequently, some vaccines against MP utilize monoclonal antibodies targeting the P1 protein to reduce its gliding speed. The P1 adhesin protein on the surface of MP is its adhesin and major antigen, containing specific T-cell and B-cell epitopes (24). After infection with MP, adults experience lymphocytopenia, with fewer T cells and B cells binding to the P1 protein compared to children. This results in the production of fewer inflammatory cytokines, thus avoiding excessive inflammatory responses. On the other hand, MP can induce immune adhesion, metabolic exhaustion, and accelerated apoptosis of lymphocytes (25). In children, the relative preservation or even elevation of peripheral lymphocyte counts during MP infection has been noted, which may reflect a vigorous but poorly regulated adaptive response.

Azithromycin has long been the first-line treatment for children with MPP. In this study, the proportion of children using azithromycin was significantly higher than that of other antibiotics and their hospitalization duration was relatively longer. Previous study suggested that drug-resistant MPP patients had a longer course of disease (26). A study in South Korea (27) and a study in China (28) indicated that the resistance rate to macrolide antibiotics in children with MPP is high. Children with MPP involving the A2063G mutation had longer disease courses and treatment durations compared to non-mutated patients. The mutation rate in children was significantly higher than in adults. This suggests that clinicians should consider the current resistance of children to macrolide antibiotics when selecting antibiotics for pediatric MPP patients. The increased resistance to macrolide antibiotics in children contribute to the shorter average hospitalization duration in adults compared to children. In the setting of high macrolide resistance rates, particularly in Asia, more judicious antibiotic selection and efforts to minimize the duration of antibiotic use are imperative.

Studies have indicated that although CRP levels in MPP patients are not markedly elevated, they are higher in the severe group compared to the mild group (29). This suggests that CRP can reflect the severity of MPP. CRP may serve both as a passive systemic marker and an active pro-inflammatory mediator (30). In MPP, CRP is predominantly synthesized by hepatocytes in response to interleukin-6 (IL-6) released from MP-infected respiratory epithelial cells and activated macrophages (31). Beyond reflecting the intensity of the innate inflammatory response, CRP itself can contribute to tissue damage by activating the classical complement pathway and inducing pro-inflammatory cytokine production in alveolar macrophages (32, 33). In this study, the combination of CRP, LYM percentage and pre-existing diseases can jointly predict the likelihood of developing severe MPP in children. And CRP was the only independent predictor of severity MPP in adults. A plausible explanation is that in adults, where the adaptive immune response is more restrained and lymphocyte dynamics are less variable, the systemic inflammatory burden measured by CRP dominates the severity landscape, making it a sufficient single predictor (34), this finding is consistent with the results reported by Yue et al. (8). In children, the interplay between the innate inflammatory signal (CRP) and the dysregulated adaptive response (lymphocyte percentage) is more critical, thus requiring a composite marker approach (35). This distinction underscores the importance of age-tailored prognostic models and suggests that CRP in adult MPP may be closer to a mediator of severity, while in children it functions primarily as a reflection of a broader, dysregulated host response (36).

## Conclusion

Unlike infections caused by other pathogens, the peculiarities of MP—such as its adherence to host cells and gliding motility—along with children’s poorer ciliary movement, narrower airways, and relatively unaffected lymphocyte count, result in more severe clinical manifestations and longer recovery times in pediatric patients compared to adults. The combination of CRP, LYM percentage and pre-existing diseases can jointly predict the likelihood of developing severe MPP in children. And CRP was the only independent predictor of severity MPP in adults. While these findings provide clinically relevant insights into the age-dependent features of MPP in the post-pandemic era, their generalizability is limited by the single-center design and the small number of severe adult cases. This study only captured patients who were sufficiently ill to require hospitalization, thereby excluding mild, community-managed cases. This may have inflated the observed severity rates.

## Data Availability

The raw data supporting the conclusions of this article will be made available by the authors, without undue reservation.
