# Supplementary material for: Comparing differences in onset and clinical manifestations between children and adults with Mycoplasma pneumoniae pneumonia and analyzing risk factors for severe cases, during the post-COVID-19 pandemic era
Source: Front Public Health. 2026 Jun 12;14:1771434. doi: 10.3389/fpubh.2026.1771434 (PMC13303955; doi:10.3389/fpubh.2026.1771434)
Supplement: Supplementary file 1 [file Table_1.DOCX]

Contents

severity classification criteria 1

Supplementary tables 2

Table S1. Comparing cumulative improvement rates between “Child” and “Adult” groups after PSM 2

Table S2. Multivariate analysis of factors influencing severe cases in “Child” and “Adult” groups 2

Table S3. Subgroup Analysis of Disease Severity by Age in Children 3

**The detailed severity classification criteria**

In children, severe MPP was defined according to the "Guidelines for Diagnosis and Treatment of Mycoplasma pneumoniae Pneumonia in Children (2023 Edition)" [reference 9]. A case was classified as severe if it met any of the following criteria: (1) persistent high fever (≥39°C) for ≥5 days or fever lasting ≥7 days without evidence of defervescence; (2) dyspnea, tachypnea (respiratory rate ≥40 breaths/min for children aged 1–5 years, or ≥30 breaths/min for children aged >5 years), hypoxemia (pulse oxygen saturation ≤93% on room air), or cyanosis; (3) respiratory distress including nasal flaring, three-depression sign, or grunting; (4) altered mental status such as lethargy, irritability, or confusion; (5) refusal to feed or poor oral intake with signs of dehydration; (6) chest imaging showing multilobar involvement, large consolidations (≥2/3 of a lobe), pleural effusion, or progressive radiographic deterioration; (7) extrapulmonary complications involving the heart, central nervous system, skin, or hematologic system; and (8) hypotension requiring fluid resuscitation or vasopressor support. Two clinicians independently reviewed and validated the severity classification based on these criteria.

In adults, severe MPP was defined according to the "Guidelines for the Diagnosis and Treatment of Community-Acquired Pneumonia in Adults" [10]. A patient was classified as severe if they met at least one major criterion (invasive mechanical ventilation requirement, or septic shock requiring vasopressors despite adequate fluid resuscitation) or at least three minor criteria (respiratory rate ≥30/min, PaO₂/FiO₂ ratio ≤250, multilobar infiltrates, confusion/disorientation, blood urea nitrogen ≥7.14 mmol/L, leukopenia [white blood cell count <4 × 10⁹/L], thrombocytopenia [platelet count <100 × 10⁹/L], hypothermia [core temperature <36°C], or hypotension requiring aggressive fluid resuscitation). Two clinicians independently reviewed and validated the severity classification based on these criteria.

**Supplementary Table 1.**Comparing cumulative improvement rates between “Child” and “Adult” groups after PSM

| Cumulative improvement rate N(%) | Child  (n=69) | Adult  (n=46) | *P value* |
| --- | --- | --- | --- |
| DAY3 | 0(0.00) | 2(4.35) | 0.081 |
| DAY4 | 1(1.45) | 12(26.09) | <0.001 |
| DAY5 | 6(8.70) | 20(43.48) | <0.001 |
| DAY6 | 10(14.49) | 20(43.48) | 0.001 |
| DAY7 | 22(31.88) | 34(73.91) | <0.001 |
| DAY8 | 29(42.03) | 38(82.61) | <0.001 |
| DAY9 | 38(55.07) | 40(86.96) | <0.001 |
| DAY10 | 49(71.01) | 41(89.13) | 0.021 |
| DAY11 | 53(76.81) | 44(95.65) | 0.006 |
| DAY12 | 55(79.71) | 44(95.65) | 0.016 |
| DAY13 | 61(88.41) | 46(100.00) | 0.017 |
| DAY14 | 64(92.75) | 46(100.00) | 0.062 |
| DAY15 | 66(95.65) | 46(100.00) | 0.152 |
| DAY16 | 68(98.55) | 46(100.00) | 0.412 |

**Supplementary Table 2.** Multivariate analysis of factors influencing severe cases in “Child” and “Adult” groups

|  | OR | 95%CI | *P* value |
| --- | --- | --- | --- |
| Child |  |  |  |
| Age | 1.008 | 0.902-1.126 | 0.890 |
| Pre-existing diseases | 5.426 | 1.214-24.263 | 0.027 |
| Thermal spike | 1.033 | 0.938-1.138 | 0.508 |
| LYM | 0.883 | 0.798-0.978 | 0.017 |
| NLR | 0.743 | 0.510-1.083 | 0.123 |
| CRP | 1.015 | 1.000-1.029 | 0.044 |
| Albumin | 1.013 | 0.988-1.038 | 0.319 |
| Adult |  |  |  |
| CRP | 1.016 | 1.003-1.028 | 0.014 |
| D-dimer | 1.213 | 0.762-1.932 | 0.416 |
|  |  |  |  |

**Supplementary Table 3.** Subgroup Analysis of Disease Severity by Age in Children

| 0-6 years | Mild(n=59) | Severe(n=10) | χ2/t | *P* value |
| --- | --- | --- | --- | --- |
| Age, years | 4.01(3.02, 5.01) | 5.00(2.07, 5.05) | 0.049 | 0.961 |
| Male, N(%) | 27(45.76) | 4(40.00) | 0.115 | 0.735 |
| BMI | 15.15(14.06, 16.26) | 14.77(14.49, 15.37) | -0.441 | 0.661 |
| Pre-existing diseases, N(%) | 3(5.08) | 2(20.00) | 2.83 | 0.093 |
| Thermal spike, ℃ | 38.80(38.13, 39.15) | 40.00(38.80, 40.75) | -3.941 | <0.001 |
| WBC, x10^9^/L | 7.11(5.96, 9.67) | 6.99(5.18, 10.09) | 0.306 | 0.761 |
| LYM, % | 32.60(24.08, 40.53) | 23.00(17.55, 31.92) | 2.034 | 0.046 |
| N,% | 57.65(47.85, 68.03) | 67.30(53.67, 73.30) | 0.073 | 0.942 |
| NLR | 1.85(1.16, 2.99) | 2.72(1.79, 4.22) | -0.135 | 0.893 |
| CRP, mg/L | 7.15(2.89, 14.63) | 20.58(5.18, 40.13) | -0.888 | 0.378 |
| ALT, IU/L | 13.00(10.00, 16.45) | 13.30(11.65, 16.50) | -1.8 | 0.076 |
| AST, IU/L | 31.10(27.35, 35.00) | 29.60(28.55, 46.40) | -1.183 | 0.241 |
| Albumin, g/L | 42.30(39.53, 44.35) | 40.00(34.50, 43.00) | 2.31 | 0.024 |
| Cr, μmol/L | 32.50(28.08, 38.00) | 28.60(25.60, 38.75) | 0.416 | 0.679 |
| PCT, ng/ml | 0.16(0.10, 0.23) | 0.17(0.10, 0.23) | 0.092 | 0.927 |
| ESR, mm/h | 30.80(30.81, 30.90) | 30.81(6.50, 30.95) | 1.833 | 0.071 |
| LDH, IU/L | 347.10(309.70, 389.48) | 443.20(306.45, 552.05) | -2.456 | 0.017 |
| D-dimer, mg/L | 0.78(0.29, 0.78) | 0.78(0.43, 1.34) | -2.934 | 0.005 |
| PT, s | 12.60(11.83, 12.60) | 12.60(11.60, 12.85) | 0.091 | 0.928 |
| tree-in-bud pattern, N(%) | 0(0.00) | 0(0.00) | - | - |
| Cumulative double lungs, N(%) | 35(59.32) | 9(90.00) | 3.483 | 0.062 |
| pulmonary consolidation, N(%) | 4(6.78) | 0(0.00) | 0.72 | 0.396 |
| Ground-glass opacity, N(%) | 4(6.78) | 0(0.00) | 0.72 | 0.396 |
| Spots/patches, N(%) | 44(74.58) | 8(80.00) | 0.135 | 0.713 |
| Strip-like patterns, N(%) | 0(0.00) | 0(0.00) | - | - |
| hydrothorax, N(%) | 3(5.08) | 1(10.00) | 0.378 | 0.539 |
| Lung mini-nodules, N(%) | 5(8.47) | 0(0.00) | 0.914 | 0.339 |
| 7-12 years | Mild(n=89) | Severe(n=8) | χ2/t | *P* value |
| Age, years | 7.09(7.01, 9.03) | 6.56(6.04, 7.77) | 1.53 | 0.129 |
| Male, N(%) | 56(62.92) | 4(50.00) | 0.519 | 0.471 |
| BMI | 15.34(14.11, 17.49) | 16.84(13.59, 17.72） | 0.197 | 0.844 |
| Pre-existing diseases, N(%) | 1(1.12) | 0(0.00) | 0.091 | 0.763 |
| Thermal spike, ℃ | 39.00(38.50, 39.45) | 40.00(39.55, 40.15) | -3.783 | <0.001 |
| WBC, x10^9^/L | 7.02(6.16, 8.58) | 7.14(5.50, 10.04) | -0.563 | 0.591 |
| LYM, % | 25.40(21.40, 30.90) | 22.25(15.88, 25.88) | 0.875 | 0.384 |
| N,% | 65.10(58.25, 70.20) | 70.15(64.33, 76.25) | -2.218 | 0.029 |
| NLR | 2.80(2.04, 3.14) | 3.16(2.49, 4.85) | -1.186 | 0.273 |
| CRP, mg/L | 8.69(4.51, 18.26) | 13.17(5.95, 32.63) | -1.058 | 0.293 |
| ALT, IU/L | 14.00(11.00, 18.00) | 14.70(11.33, 19.50) | -0.048 | 0.962 |
| AST, IU/L | 27.00(23.50, 31.00) | 29.55(23.75, 34.08) | -1.03 | 0.306 |
| Albumin, g/L | 41.00(38.75, 42.60) | 42.05(38.60, 46.23) | -1.016 | 0.344 |
| Cr, μmol/L | 41.60(38.50, 45.85) | 40.95(39.28, 45.53) | -0.114 | 0.909 |
| PCT, ng/ml | 0.23(0.10, 0.23) | 0.23(0.16, 0.26) | 0.094 | 0.925 |
| ESR, mm/h | 30.81(30.81, 30.81) | 30.81(30.81, 37.70) | -0.419 | 0.676 |
| LDH, IU/L | 290.20(263.55,330.50) | 346.05(317.13,391.73) | -2.281 | 0.025 |
| D-dimer, mg/L | 0.76(0.22, 0.78) | 0.32(0.21, 0.46) | 0.5 | 0.618 |
| PT, s | 12.60(12.40, 12.80) | 13.15(12.35, 14.30 | -1.464 | 0.185 |
| tree-in-bud pattern, N(%) | 0(0.00) | 0(0.00) | - | - |
| Cumulative double lungs, N(%) | 42(47.19) | 6(75.00) | 2.271 | 0.132 |
| pulmonary consolidation, N(%) | 16(17.98) | 3(37.50) | 1.776 | 0.183 |
| Ground-glass opacity, N(%) | 6(6.74) | 0(0.00) | 0.575 | 0.448 |
| Spots/patches, N(%) | 57(64.04) | 6(75.00) | 0.387 | 0.534 |
| Strip-like patterns, N(%) | 3(3.37) | 1(12.50) | 1.547 | 0.214 |
| hydrothorax, N(%) | 2(2.25) | 1(12.50) | 2.574 | 0.109 |
| Lung mini-nodules, N(%) | 11(12.36) | 0(0.00) | 1.115 | 0.291 |
| 13-17 years | Mild(n=22) | Severe(n=12) | χ2/t | *P* value |
| Age, years | 13.08(12.08, 15.25) | 16.00(14.29, 16.00) | -2.995 | 0.005 |
| Male, N(%) | 13(59.09) | 6(50.00) | 0.26 | 0.61 |
| BMI | 19.10(17.35, 23.08) | 19.03(16.36, 19.90) | 1.202 | 0.238 |
| Pre-existing diseases, N(%) | 2(9.09) | 2(16.67) | 0.429 | 0.512 |
| Thermal spike, ℃ | 39.00(39.00, 39.20) | 39.60(40.00, 40.88) | -5.394 | <0.001 |
| WBC, x10^9^/L | 7.03(5.90, 9.20) | 6.28(3.95, 8.50) | 0.484 | 0.631 |
| LYM, % | 20.25(17.22, 25.90) | 18.70(14.95, 24.05) | 0.958 | 0.345 |
| N,% | 70.10(63.85, 76.15) | 72.45(68.00, 78.30) | -1.457 | 0.155 |
| NLR | 3.32(2.46, 3.95) | 3.95(2.56, 4.92) | -0.957 | 0.346 |
| CRP, mg/L | 18.93(15.34, 28.20) | 39.75(15.51, 72.16) | -1.599 | 0.12 |
| ALT, IU/L | 15.00(12.00, 26.03) | 15.15(12.25, 27.48) | 0.236 | 0.815 |
| AST, IU/L | 22.00(20.00, 25.38) | 21.00(17.25, 30.25) | 0.706 | 0.486 |
| Albumin, g/L | 40.90(37.90, 41.95) | 41.80(39.85, 42.55) | -1.319 | 0.196 |
| Cr, μmol/L | 57.00(51.43, 68.75) | 57.65(51.13, 64.45) | 0.286 | 0.777 |
| PCT, ng/ml | 0.11(0.10, 0.23) | 0.23(0.10, 0.28) | -1.923 | 0.063 |
| ESR, mm/h | 30.81(30.81, 30.85) | 30.81(30.81, 34.25) | 0.124 | 0.902 |
| LDH, IU/L | 261.10(235.15, 308.72) | 265.85(237.68, 302.58) | 0.798 | 0.431 |
| D-dimer, mg/L | 0.73(0.49, 0.92) | 0.35(0.19, 0.76) | 0.395 | 0.695 |
| PT, s | 12.95(12.60, 13.63) | 12.50(12.23, 13.73) | 0.748 | 0.46 |
| tree-in-bud pattern, N(%) | 1(4.55) | 0(0.00) | 0.562 | 0.453 |
| Cumulative double lungs, N(%) | 8(36.36) | 4(33.33) | 0.031 | 0.86 |
| pulmonary consolidation, N(%) | 4(18.18) | 2(16.67) | 0.012 | 0.912 |
| Ground-glass opacity, N(%) | 1(4.55) | 0(0.00) | 0.562 | 0.453 |
| Spots/patches, N(%) | 18(81.82) | 8(66.67) | 0.991 | 0.32 |
| Strip-like patterns, N(%) | 1(4.55) | 1(8.33) | 0.201 | 0.654 |
| hydrothorax, N(%) | 2(9.09) | 0(0.00) | 1.159 | 0.282 |
| Lung mini-nodules, N(%) | 3(13.64) | 1(8.33) | 0.21 | 0.646 |
